# Does surgery followed by physiotherapy improve short and long term outcome for patients with atraumatic shoulder instability compared with physiotherapy alone? - protocol for a randomized controlled clinical trial

**DOI:** 10.1186/1471-2474-15-439

**Published:** 2014-12-17

**Authors:** Anju Jaggi, Susan Alexander, Robert Herbert, Lennard Funk, Karen A Ginn

**Affiliations:** Royal National Orthopaedic Hospital NHS Trust, Stanmore, UK; Neuroscience Research Australia, Randwick, Australia; Wrightington, Wigan & Leigh NHS Foundation Trust, Wrightington Hospital, Wrightington, UK; Discipline of Biomedical Science, Sydney Medical School, The University of Sydney, Sydney, Australia

**Keywords:** Shoulder instability, Randomized controlled trial, Stabilization surgery, Capsular plication, Physiotherapy, Musculoskeletal

## Abstract

**Background:**

Shoulder instability is a common problem affecting young adults. Stabilization surgery followed by physiotherapy rehabilitation has been shown to reduce the chance of further episodes of shoulder dislocation and to improve quality of life in patients who sustain a shoulder dislocation as a result of a high collision trauma, but it is unclear if surgical intervention is beneficial for patients with atraumatic shoulder instability who have structural damage at the shoulder. The aim of this randomized controlled clinical trial is to determine if the addition of surgical intervention to physiotherapy rehabilitation improves outcomes for patients with atraumatic shoulder instability who have sustained soft tissue damage at their joint.

**Methods/Design:**

140 participants will be recruited. Patients with feelings of insecurity (apprehension) at their shoulder joint, which is not the result of a collision injury, with physical signs of shoulder joint instability will be invited to participate. Consenting participants will undergo arthroscopic investigation of the shoulder joint. Patients with capsulolabral damage will be randomly allocated using a concealed allocation procedure to either stabilization surgery immediately following the arthroscopic examination or no additional surgical procedure. All participants will then receive the same postoperative physiotherapy protocol for up to 6 months. Outcomes (pain, functional impairment and number of shoulder dislocations sustained) will be evaluated prior to surgery and, together with participant-reported improvement, again at 6, 12 and 24 months after randomization. The primary endpoint will be pain and functional impairment at 2 years. Participants, clinical staff (but not surgeons) and assessors will be blind to whether stabilization surgery was performed. Data analysis will be conducted on an intention-to-treat basis with the focus on estimation of the effect.

**Discussion:**

This trial will have a direct and immediate impact on clinical decision making by establishing if patients presenting with soft tissue shoulder damage associated with atraumatic shoulder instability should be referred for stabilization surgery before commencing physiotherapy rehabilitation in order to ensure optimal outcome. This in turn will ensure effective, efficient use of scarce health resources to manage this common often disabling musculoskeletal condition.

**Trial registration:**

Study was registered with National Institutes of Health Clinical Trials Protocol Registration System in December 2012.

ClinicalTrials.gov Identifier: NCT01751490.

## Background

The shoulder is the most mobile joint in the body and the joint most susceptible to instability. More than any other joint, the shoulder relies on dynamic muscle support to achieve functional stability [1].

Shoulder instability is defined as symptomatic, abnormal motion at the shoulder (glenohumeral) joint. It can result in a spectrum of symptoms including pain, a feeling of insecurity, and dislocation [2]. Shoulder instability is a common problem predominantly occurring in the younger population [3]. Patients suffering from shoulder instability experience significant reduction in ability to function physically, socially and emotionally [4]. The resulting decrease in quality of life ranks in severity with major medical conditions such as hypertension, congestive cardiac failure, acute myocardial infarction, diabetes mellitus and clinical depression [4].

Shoulder instability can occur as a result of shoulder trauma, e.g. following dislocation (traumatic instability) or it can develop without a traumatic episode (atraumatic instability). A combination of factors can result in shoulder instability: damage to the joint (e.g. bony Bankart lesion of the glenoid fossa, Hills Sachs lesion of the humeral head); damage to the capsulolabral complex (e.g. tears/detachment of the glenoid labrum, joint capsule or ligaments); and loss of dynamic muscle control. Treatment for shoulder instability consists of surgery to repair bony and capsulolabral damage and physiotherapy to restore muscle control mechanisms. However, few high quality randomized controlled clinical trials have investigated which combination of operative and non-operative treatment affords the most effective outcome for patients presenting with shoulder instability.

Management of shoulder instability typically consists of a combination of immobilization in supportive slings, surgical intervention to repair or tighten damaged capsular, ligamentous and/or labral structures in the shoulder joint, and physiotherapy rehabilitation including strengthening exercises [5] and exercises to restore normal dynamic stabilization mechanisms at the shoulder. Currently, the management of shoulder instability differs depending on the precipitating factors. Operative intervention followed by physiotherapy rehabilitation is the preferred option for instability that has occurred as a result of trauma. Non-operative/conservative treatment is preferred as the initial management of atraumatic instability [6]. However, these practices are not based on rigorous evidence. Few high quality randomized controlled clinical trials have investigated which combination of operative and non-operative treatment affords the most effective outcome for patients presenting with shoulder instability.

Evidence from one randomized controlled clinical trial [7] and several prospective cohort studies [8–10] comparing surgery followed by rehabilitation to rehabilitation alone provides consistent support in favour of operative intervention in patients following a traumatic primary shoulder dislocation resulting in bony and/or capsulolabral damage. Two to three years after injury young adults (<30 years of age) who had received the surgical option demonstrated significant reduction in re-dislocation rate [7, 10] and a small but clinically significant improvement in quality of life scores [7]. In addition, young military cadets demonstrated a significant reduction in the development of recurrent instability [8, 9].

No randomized clinical trials or prospective cohort studies have compared operative and non-operative treatment in the management of atraumatic shoulder instability. Evidence from retrospective observational studies suggests that both surgical and non-surgical treatment may be effective. Several studies indicate that an excellent outcome can be achieved with specialist physiotherapy treatment for atraumatic shoulder instability [11, 12]. Other studies indicate that surgery is as effective for atraumatic as for traumatic instability [13, 14], can result in a decreased shoulder dislocation recurrence rate [15] and can result in improvement in function, stability and motion [16].

On the other hand, some studies indicate that surgery is not successful for the treatment of atraumatic shoulder instability and can result in longer term detrimental effects. A small prospective observational study did not find significant functional improvement in young athletes with multidirectional instability following surgery [17] and failure rates of up to 60% have been reported following thermal capsulorraphy surgery [18]. In addition, there is some evidence that patients who have undergone unsuccessful surgery for atraumatic instability and subsequently proceed with conservative rehabilitation have worse outcomes than those who are initially treated conservatively [6]. It has also been suggested that in younger patients whose atraumatic instability is primarily due to abnormal, unbalanced muscles forces on the shoulder joint surgical intervention may contribute to early degenerative changes. [19, 20].

In summary, the limited evidence available suggests that, in patients with traumatic shoulder instability, surgical intervention followed by physiotherapy rehabilitation is the most effective management strategy but in patients with atraumatic instability without structural damage to shoulder capsule or ligaments, surgery is not helpful or detrimental. The role of surgical intervention in patients with atraumatic shoulder instability associated with capsulolabral damage is still unclear. A robust clinical trial is required to determine if surgical intervention is necessary to provide optimal short, medium and longer term outcomes for these patients.

### Study objectives

The aim of this randomized controlled clinical trial is to determine whether surgical intervention followed by physiotherapy rehabilitation improves pain and functional impairment outcomes in patients suffering from atraumatic shoulder instability associated with capsulolabral damage, compared to physiotherapy alone. We hypothesized that the patients receiving stabilization surgery followed by post-operative physiotherapy rehabilitation would have significantly greater short and long term improvement in pain and function.

## Methods/Design

### Design

A two-arm, double-blind randomized controlled clinical trial will be conducted. 140 patients will be recruited in two study centres and will undergo a clinical assessment to assess potential eligibility for the study. Potentially eligible patients who consent to participate will undergo arthroscopy and those with arthroscopic evidence of capsulolabral damage will be randomly allocated during the procedure into one of two groups: a *stabilization surgery* group and a *control* group. The primary outcome (pain and functional impairment) and secondary outcomes (participant-reported improvement and number of shoulder dislocations sustained) will be evaluated at baseline and 6, 12 and 24 months after randomization. Additional secondary outcomes of shoulder rotation range of motion and strength will be evaluated at 6 months after randomization. Ethics approval to conduct this study has been granted by the NRES Committee London-Stanmore and NRES Committee Wrightington, Wigan and Leigh.

### Participants

Patients over 18 years of age of all activity levels will be eligible to participate if they report insecurity (apprehension) at the shoulder joint, have physical signs of shoulder instability (provocation of apprehension with drawer and apprehension tests), and have evidence of capsulolabral damage in the shoulder joint on arthroscopic examination. Damage to the capsulolabral complex includes any splits, fraying or detachment of any region of the glenoid labrum including the superior labrum. Patients will be excluded if they have a history of a high collision shoulder injury precipitating their apprehension symptoms, evidence of bony injury around the glenoid rim and/or humeral head or rotator cuff tear on arthroscopic examination, neural damage affecting the upper limb, or previous shoulder surgery. Prior physiotherapy treatment will not exclude eligible patients from participating in this clinical trial.

Potential participants will be identified from patients attending clinics conducted at the participating hospitals. Experienced clinicians in these clinics will inform potential participants of the clinical trial and provide them with information regarding the trial in the form of a written information sheet. With the patient’s permission this clinician will forward contact information to the research team. An experienced research assistant, who will be a registered health professional, will contact the potential participant to discuss the purpose of the study and to answer any questions this patient may have regarding the study. The research assistant will them seek written consent from those who volunteer to participate.

### Interventions

140 participants will be recruited and randomly allocated to either a:

1. stabilization surgery group (70 participants). Participants in this group will receive an arthroscopic investigation of the shoulder joint followed by arthroscopic stabilization surgery and postoperative physiotherapy rehabilitation.
2. control group (70 participants). Participants in this group will receive an arthroscopic investigation of the shoulder joint followed by postoperative physiotherapy rehabilitation.

#### Arthroscopic examination

All potentially eligible patients will undergo arthroscopic examination of the shoulder under general anaesthetic. Arthroscopy is the gold standard for diagnosing subtle capsulolabral lesions at the shoulder [21, 22]. The patient will be prepped and draped on the operating table in the beach chair position. A 1 cm incision will be made over the back of the shoulder joint and a metal trochar used to penetrate the joint capsule. An arthroscope will be introduced into the joint. Another 1 cm incision will be made over the anterior aspect of the joint and a metal probe introduced into the joint to palpate and move intra-articular structures in order to fully assess the joint. The arthroscope will then be replaced through the front of the joint in order to visualize the posterior joint structures.

Following confirmation that the patient is eligible to participate in the trial surgery theatre staff will open a pre-prepared sealed envelope identifying group allocation for each participant and inform the surgeon if stabilization is to proceed. To ensure blinding of the participants, physiotherapy staff associated with post-operative care and research assistants, the surgeon and theatre staff will be asked not to reveal group allocation (i.e., whether stabilization surgery was performed) to anyone associated with the study.

#### Stabilization surgery

Participants allocated to the *stabilization surgery group* will undergo capsular plication and labrum repair surgery as appropriate. Capsular plication surgery will involve the placement of suture anchors into the bony glenoid with sutures passed through the shoulder joint capsule to secure the redundant capsule to the glenoid fossa, effectively ‘tightening’ the joint. Labrum repair will be performed using standard suture anchors incorporating the plication where appropriate. All participants will receive the same post-operative clinical care from the surgical team to monitor progress and deal with any complications that may arise. Standard care includes review at 1, 3 and 6 months post surgical examination/intervention.

#### Physiotherapy

All participants will receive the same post-operative physiotherapy protocol aimed at improving shoulder muscle function based on a pre-prepared treatment algorithm. All participants will be immobilized in a sling for four weeks following the surgical procedure but will be allowed to perform controlled scapular and glenohumeral joint movements within ranges which would not compromise a capsulolabral repair. Thus restrictions on post-operative treatment to protect the surgical procedure will apply to all participants. Thereafter, the aim of physiotherapy treatment will be to improve the function of the rotator cuff muscles by an active home-based exercise program. The treatment algorithm allows the physiotherapist to choose specific exercises directed to the rotator cuff or to improve their function by incorporating exercises which involve the entire kinetic chain. To ensure accurate exercise performance multimodal feedback (visual, biofeedback, taping) can be utilized. The type, load and frequency of exercises will be individually tailored to the needs of each participant by the physiotherapist who will monitor and upgrade the exercises as rotator cuff muscle function improves. A maximum of 12 treatment sessions with the physiotherapist over a maximum period of six months after surgery will be conducted.

### Outcome measures

#### Primary outcome measure

Pain and functional impairment, measured using the Western Ontario Shoulder Instability Index (WOSI), will be the primary outcome. The WOSI is a disease specific, self-administered questionnaire consisting of 21 items assessing the physical symptoms associated with shoulder instability (10 items) as well as the impact of shoulder instability on sport/recreation/work (4 items), lifestyle (4 items) and emotional state (3 items). It was rigorously developed using patient input, demonstrates good construct validity and excellent reliability and is responsive to change [3].

#### Secondary outcome measures

Data on four secondary outcomes will be collected:

1. global perceived effect assessing participant-reported improvement measured on an 11 point scale from −5 (“vastly worse”) to +5 (“completely recovered”) [23].
2. active external and internal shoulder rotation range of motion measured using digital photography [24–26] This method was chosen over goniometry as it is less likely to exacerbate shoulder symptoms due to the shorter time required to complete measurements.
3. isometric external and internal shoulder rotation strength measured using a hand-held dynamometer which has been shown to exhibit acceptable reliability when tested on patients with strength deficits [27]
4. number of episodes of post-operative shoulder dislocation. An episode of shoulder dislocation will be defined as separation of the articular surfaces of the shoulder joint which require assistance to be relocated.

All outcome measurements will be re-assessed 6 months after randomization by a research assistant who will be blind to treatment group allocation. At 12 and 24 months after randomization pain and functional impairment, participant-reported improvement and number of shoulder dislocations sustained will again be re-assessed by a blinded assessor.

### Sample size

The sample size was calculated assuming a 10.4% change on the WOSI given a standard deviation of 18% change, a significance level of 5% and assuming a 10% (worst-case) loss to follow-up. A 10.4% change in total WOSI score represents the minimally important change required to represent a clinically significant improvement [28]. This is a conservative sample size estimate as it ignores the additional power conferred by the longitudinal analysis that is to be used in this study.

### Sequence generation

An allocation assignment schedule will be prepared prior to commencement of the clinical trial using random numbers generated with the ralloc command in the Stata statistical package [29]. The random allocation schedule will be prepared by a member of the research team not associated with identification of potential participants or any aspects of recruitment, treatment or assessment of participants, and will remain concealed until the analysis is complete. Allocation will be in randomly permuted blocks.

### Adverse events

Participants in this clinical trial will be subjected to the risks associated with orthopaedic surgery under general anaesthesia. However, participation in this clinical trial will not entail additional risks beyond those associated with standard care options for atraumatic shoulder instability at the clinical sites involved in the study. Information regarding adverse events will be monitored throughout this clinical trial and reported on a regular basis to the Trial Management Group and Steering Committee associated with the Stanmore Clinical Research Centre. Both closed and open questioning will be utilized to seek information from participants and physiotherapy staff involved in post-operative treatment, all of whom will be unaware of whether stabilization surgery was performed.

### Statistical analyses

#### Interim analysis

One interim analysis of efficacy will be conducted when 50% of the estimated sample has completed one year follow-up assessment. The decision to continue the trial or stop the trial early will be made by the Trial Management Group and Steering Group, informed by an analysis of the Haybittle-Peto boundary [30]. The p-value threshold for this interim analysis will be set at 0.001. The final analysis will be evaluated at the significance level specified in the sample size calculation i.e. p < 0.05.

#### Primary analyses

The primary analyses will be conducted on an intention-to-treat basis. The focus will be on estimation of the effect [31] at 2 years, but hypothesis tests will also be conducted. Between-group comparisons will be conducted using a linear model in which the outcome is a linear function of a dummy-coded variable representing group membership (stabilization surgery or control group) and baseline score [32]. For the outcomes of pain and functional impairment, participant-reported improvement and incidence of shoulder dislocations, all of which are measured at baseline and 6, 12 and 24 months, mixed models will be used. The mixed models will incorporate a random intercept for subject. A negative binomial model will be used to analyze data on the incidence of dislocations. A sensitivity analysis will be conducted in which multiple imputation is used to account for missing data [33, 34].

## Discussion

Shoulder instability is a common problem affecting young adults which can result in a significant decrease in quality of life. Stabilization surgery followed by physiotherapy rehabilitation has been shown to reduce the chance of further episodes of shoulder dislocation and to improve quality of life scores in patients who sustain a shoulder dislocation as a result of a high collision trauma which results in significant bony damage to the shoulder joint. On the other hand, experts believe that surgical intervention is detrimental in patients with recurrent shoulder instability who do not exhibit structural damage in the joint. For patients with atraumatic shoulder instability who do have soft tissue damage at the shoulder joint, it is unclear if surgical intervention is beneficial as no randomized clinical trials comparing operative and non-operative management have been conducted. Several studies indicate that an excellent outcome can be achieved with specialist physiotherapy treatment alone. However, it is not known if the addition of stabilization surgery to physiotherapy rehabilitation would deliver superior long term outcomes in this cohort of patients.

The aim of this randomized controlled clinical trial is to determine if the addition of surgical intervention to physiotherapy rehabilitation is necessary to provide optimal outcomes for the cohort of patients with atraumatic shoulder instability who exhibit capsulolabral joint damage. In order to address any placebo effect that may be associated with the high levels of stress and the rituals associated with surgery this clinical trial includes a sham-controlled surgical arm [35].

The results of this study will have direct and immediate impact on clinical decision making by establishing definitively if patients presenting with soft tissue shoulder joint damage associated with atraumatic shoulder instability should be referred for stabilization surgery before commencing physiotherapy rehabilitation in order to ensure optimal outcome. This in turn will ensure effective, efficient use of scarce health resources to manage for this common, often disabling, musculoskeletal condition.

### Ethical approval

The NRES Committee London – Stanmore provided ethical approval for this study in October 2012. REC Reference number 12/LO/1501.
